# Diffusion tensor imaging and plasma immunological biomarker panel in a rat traumatic brain injury (TBI) model and in human clinical TBI

**DOI:** 10.3389/fimmu.2023.1293471

**Published:** 2024-01-08

**Authors:** Xuan Vinh To, Abdalla Z. Mohamed, Paul Cumming, Fatima A. Nasrallah

**Affiliations:** ^1^ The Queensland Brain Institute, The University of Queensland, Queensland, Australia; ^2^ Thompson Institute, University of the Sunshine Coast, Queensland, Australia; ^3^ Department of Nuclear Medicine, Bern University Hospital, Bern, Switzerland; ^4^ School of Psychology and Counselling, Queensland University of Technology, Brisbane, Queensland, Australia; ^5^ The Centre for Advanced Imaging, The University of Queensland, Queensland, Australia

**Keywords:** traumatic brain injury, controlled cortical impact model, inflammatory markers, diffusion tensor imaging, fractional anisotropy

## Abstract

**Introduction:**

Neuroinflammatory reactions play a significant role in the pathology and long-term consequences of traumatic brain injury (TBI) and may mediate salutogenic processes that white matter integrity. This study aimed to investigate the relationship between inflammatory markers and white matter integrity following TBI in both a rat TBI model and clinical TBI cases.

**Methods:**

In the rat model, blood samples were collected following a controlled cortical impact (CCI) to assess a panel of inflammatory markers; MR-based diffusion tensor imaging (DTI) was employed to evaluate white matter integrity 60 days post-injury. 15 clinical TBI patients were similarly assessed for a panel of inflammatory markers and DTI post-intensive care unit discharge. Blood samples from healthy controls were used for comparison of the inflammatory markers.

**Results:**

Time-dependent elevations in immunological markers were observed in TBI rats, with a correlation to preserved fractional anisotropy (FA) in white matter. Specifically, TBI-induced increased plasma levels of IL-1β, IL-6, G-CSF, CCL3, CCL5, and TNF-α were associated with higher white matter integrity, as measured by FA. Clinical cases had similar findings: elevated inflammatory markers (relative to controls) were associated with preservation of FA in vulnerable white matter regions.

**Discussion:**

Inflammatory markers in post-TBI plasma samples are ambivalent with respect to prediction of favourable outcome versus a progression to more pervasive pathology and morbidity.

## 1 Introduction

With an estimated annual incidence of 295 per 100,000 population, traumatic brain injury (TBI) is a major contributor to mortality and morbidity across the world (1). According to an influential position statement, TBI is “an alteration in brain function, or other evidence of brain pathology, caused by an external force” (2). In the clinical setting, alteration in brain function can refer to loss of or decreased level of consciousness, loss of memory immediately before or after the injury, neurologic deficits or altered mental state and other evidence of brain pathology, which can include visual, neuroradiologic, or laboratory-confirmed damage to the brain (2). TBI severity has traditionally been classified using the Glasgow Coma Scale (GCS) (3), which mainly depends on the clinicians’ impression of the level of consciousness soon after the impact. As such, the GCS may not be representative of the diverse nature of TBI and its long-term consequences, which can include neuronal cell loss, axonal damage, demyelination, disconnection of cortical and subcortical structures, and neuroinflammation (4–7). Consequently, clinical assessment of symptoms present close to the time of injury are a poor predictor of long-time prognosis with respect to morbidity and mortality (8).

Neuroinflammation and axonal injury are considered key elements in the long-term responses to an initial TBI event. Notably, neuroinflammation is a complex phenomenon initiated post-TBI by acute damage to the blood-brain barrier (BBB), but with a complex propagation to a secondary phase that involves feedback interaction between pro- and anti-inflammatory mechanisms (9). In TBIs, neuroinflammation is neither detrimental nor beneficial *per se*, but entails detrimental processes such as neuronal cell death and neurodegeneration, as well as beneficial events such as debris clearance and neuronal regeneration (9). This link between neuroinflammation and post-TBI neural degeneration and regeneration spurred various attempts to use biological fluid markers of neuroinflammation as a prognostic indicator of TBI. Although, the dynamic evolution of immunological biomarker levels in the blood does not necessarily correlate with brain findings derived from cerebral-spinal fluid (CSF) (10–13), due to ease of access, blood immunological biomarkers have been drawing increasing interest as prognostic tools for TBI (8). Nevertheless, some early studies (10, 14–17) on neuroinflammation biomarkers in TBI often used a reductionist approach focusing on a single biomarker or oversimplifying the interactions between biomarkers. Future studies into biomarkers in TBI may benefit from using more comprehensive biomarker panels.

Traumatic axonal injuries (TAIs) and white matter damage are common pathologies in TBIs, which are often detectable using non-invasive neuroimaging techniques. Specifically, computed tomography (CT) can detect larger haemorrhagic TAIs, susceptibility-weighted magnetic resonance imaging (MRI) can detect smaller haemorrhagic TAIs, and diffusion-weighted MRI or diffusion tensor imaging (DTI) MRI can detect small non-haemorrhagic TAIs (18) and other microstructural changes in human TBIs (19–21) and experimental animal TBIs (6, 22–24). Human TBIs are complex in their origins, severities, mechanisms of injury, patients’ background, immunological reactions, and subsequent evolution of the pathologies. Given this heterogeneity, animal models of TBI such as the controlled cortical impact (CCI) model in the rat (25) provide a robust platform for translational studies with reduced inter-subject variability, while capturing key aspects of the common pathologies in human TBIs (26). DTI generates an index of fractional anisotropy (FA) of water diffusion in brain tissue, which is generally reduced in cases of white matter damage and TAIs (6, 27). Given the association of neuroinflammation with post-TBI neuropathology and the common utilisation of DTI in clinical imaging, we hypothesised that inflammatory markers in blood should bear a relation to TBI outcomes with respect to DTI quantification of TAIs. We tested this hypothesis by applying a panel of plasma inflammatory markers in the rodent CCI TBI model and in clinical TBI cases, both in relation to DTI indices of TAI.

## 2 Materials and methods

### 2.1 Study subjects and participants

The animal experiments received approval by the Animal Research Ethics Committee (AEC) of the University of Queensland (approval number: QBI/036/16/MAIC). Fourteen male Sprague-Dawley rats aged 8 to 10 weeks and weighing 300 to 340 grams were acquired from the Animal Resource Centre (ARC, Western Australia). They were kept in standard laboratory conditions with a 12-hour light-dark cycle and provided with unrestricted access to food and water. The rats were randomly assigned to sham surgery (n = 6) and TBI (n = 8) groups. Blood samples were collected from a tail vein of each rat at days 1, 3, 7, and 60 after the respective surgeries. The plasma fractions were separated and stored at -80°C until analysis using the plasma biomarker panels of cytokines. Additionally, magnetic resonance imaging (MRI) scans were performed at day 60 after the surgeries.

The human study part was approved by the institutional Human Research Ethics Committee (approval number HREC/16/QRBW/604). We obtained informed consent from conscious TBI patients or their surrogate decision-makers upon their admission to the neurosurgical unit of a level-1 trauma hospital in Brisbane, Queensland, Australia. This hospital is part of the Metro North Health network, Queensland, Australia, which serves a population of approximately 900,000. We excluded individuals who were under 18 or over 80 years old, had a history of neurodegenerative disease, significant mental health disorders, or contraindications for MRI. All patients had positive findings on their initial CT scan or met the criteria for mild TBI as defined by the National Institute of Neurological Disorders and Stroke (NINDS) (28) or the American Congress of Rehabilitation Medicine (ACRM) (29), which include documented loss of consciousness or post-traumatic amnesia following a head injury. We collected general demographic information and key clinical parameters for all participants from their respective clinical charts. These parameters included details about the mechanism of injury, the initial GCS score, and pupillary reactions at the time of admission.

To serve as a comparison group for the plasma immunological biomarker panel, we recruited 19 healthy controls who were matched in terms of demographics. The selection criteria for these controls included the absence of significant neurodegenerative or mental health disorders and no history of diagnosed TBI within the previous 12 months. The control group did not undergo MRI scans.

### 2.2 Animal TBI experiment

#### 2.2.1 Controlled cortical impact (CCI) traumatic brain injury model

The procedures for CCI are described in detail in our previous publications (24, 30). In brief, we anesthetized the rats with isoflurane (5% for induction, 1-2% for maintenance) in a 40:60 O_2_:medical air mixture at a flow rate of 2 L/min. After induction, the rats were placed in a stereotaxic frame, part of the right scalp retracted, and a 5 mm diameter craniotomy window was carefully made over the right cerebral hemisphere, centred at 2.5 mm posterior to bregma and 3 mm right lateral to the sagittal suture. In the TBI group, a CCI injury was induced using a pneumatically driven impactor (TBI 0310, Precision System and Instrumentation, USA) with a cylindrical 4 mm diameter tip, employing the following impact parameters: impact velocity = 5 m/s, penetration depth = 2 mm, and dwell time = 200 ms. For the sham animals, the craniotomy was performed similarly, but no actual impact was delivered.

Following the impact or sham procedure, the bone flap was replaced, and the scalp sutured. The total anaesthesia time was under 20 minutes. Afterwards, the animals were removed from the stereotaxic frame and placed on a heated surface for monitoring and recovery. Once the animals displayed full mobility and alertness, they were returned to their home cages. Importantly, no animals exhibited noticeable signs of motor deficits after the procedure.

#### 2.2.2 Blood sample collection and plasma immunological biomarker quantification

The details of our procedures for blood sampling and quantifying plasma immunological markers are presented in a prior publication (30). Blood samples (approximately 1mL) were collected at four timepoints (days 1, 3, 7, and 60 post-procedure) through tail vein venepuncture into EDTA collection tubes. Blood samples were briefly centrifuged to separate the plasma fraction, which was then passed through glass wool and a 0.22-micron filtration column via a second centrifugation and stored at -80 °C for further analysis. For immunological biomarker analysis, we used a rat cytokine kit from Bio-Rad (Cat# 12005641). The plasma samples were thawed, diluted, and then processed exactly according to the manufacturer’s instructions, with simultaneous detection of analytes in 96-well plates using the robotic liquid handling workstation epMotion 5075 (Eppendorf). The assay plates were washed using the Bio-Plex Pro II magnetic plate washer (Bio-Rad), and the measurements of analytes were obtained using the Bio-Plex Systems 200 (Bio-Rad). The analysis of samples and creation of reference standard curves [Log(x) - Linear(y)] were performed using the Bio-Plex Manager v6.1 software (Bio-Rad). The following markers were quantified: interleukins (IL) IL-1α, IL-1β, IL-2, IL-4, IL-5, IL-6, IL-7, IL-10, IL-12p70, IL-13, IL-17α, and IL-18, granulocyte colony-stimulating factor (G-CSF), granulocyte-macrophage colony-stimulating factor (GM-CSF), macrophage colony-stimulating factor (M-CSF), interferon gamma (IFN-γ), chemokine (C-X-C motif) ligand 1 (CXCL1), chemokine ligands (CCL) CCL2, CCL3, CCL5, CCL20, vascular endothelial growth factor (VEGF), and tumour necrosis factor alpha (TNF-α).

#### 2.2.3 Magnetic resonance imaging (MRI) data collection and processing

Details of the animal MRI procedure are presented in a prior publication (30). On day 60 after the sham or TBI procedure, anaesthesia for MRI was induced as above using 2-4% isoflurane for positioning in an MRI-compatible cradle (Bruker Biospin, Germany) equipped with ear bars and bite bars to immobilise the head. Isoflurane concentration was reduced to 1-2% for cradle positioning. During the MRI scan, we provided further anaesthesia with an intraperitoneal bolus dose of 0.1 mg/kg medetomidine, followed immediately by intraperitoneal continuous infusion of 0.1 mg/kg/h medetomidine. Isoflurane concentration was gradually reduced to the 0−0.3% range for most of the scan, aiming to maintain a respiration rate in the 60−95 breaths per minute. We applied water circulation by a pump and thermostatic heater (SC100, Thermo Scientific, USA) to warm the animal cradle, thereby maintaining the animal’s rectal temperature at 36 ± 1°C. At the end of the MR examination, we applied an intraperitoneal bolus dose of 0.1 mg/kg atipemazole (Antisedan, Pfizer, Germany) for medetomidine reversal.

As described in (23, 24), rat MRI scans were acquired on a 9.4 T Bruker system (BioSpec 94/30USR, Bruker, Germany) running the Paravision 6.0.1 software (Bruker, Germany), along with a volume transmitter coil and a four-element array receiver coil. Anatomical imaging was performed using T2-weighted (T2w) rapid-relaxation-with-enhancement (RARE) sequence with the following parameters: repetition time (TR)/Echo Time (TE) = 5900/65 ms, RARE factors = 8, number of averages = 2, FOV = 25.6 × 32 mm, matrix size = 256 × 256 × 40, and 0.5 mm-thick slices, giving an effective output spatial resolution of 0.1 × 0.125 × 0.5 mm. Diffusion-weighted images were collected using a spin-echo echo-planar imaging (EPI) sequence with TR/TE = 10000/29 ms, FOV = 24.8 × 24.8 mm, matrix size = 108 × 108 × 41, and 0.5 mm-thick slices with 0.1 mm slice gaps, giving effective output spatial resolution of 0.23 × 0.23 × 0.6 mm. A b-value of 750 s/mm^2^ and 32 diffusion-weighted directions, and 4 volumes of b=0 s/mm^2^ were used.

Rat MRI data were exported from the scanner to DICOM format and converted to NIFTI format using MRIcron (31). The MRI images were given a header file with voxel size 10 times larger than the original voxel size to enable the use of image processing tools originally developed for human brain (32). We have presented details of the processing procedures for rat MRI data in a prior publication (30). The sham animals’ structural images were used to construct a study-specific T2w structural template in the SIGMA *in vivo* rat brain template space (33). Spatial normalisations of sham and TBI rat brains to this study-specific template were performed using the constrained cost function masking (CCFM) approach, which is an extension of the original cost function masking approach (34). In our modification, the template was registered to the individual subject’s image (rather than vice versa) with an additional cost function mask that excluded non-brain structures (for the template and individual images) and the lesioned areas (for the TBI individual images) using symmetric diffeomorphic image registration with cross-correlation (SyN-CC) (35), as implemented in Advanced Normalization Tool (ANTS v.2.3.4) (36). Using the inverse of the transformation, we then warped images in the T2w structural image space to the common space.

Next, the four b=0 volumes were averaged to generate a b=0 spatial representation of the diffusion MRI data, which were then corrected for motion and eddy current using FSL’s eddy_correct, and then registered to the T2w structural image using ANTS SyN-CC registration. The warping field obtained from this step was combined with the inverse warping field obtained from the template to individual T2w structural image registration step to enable the warping of images in the diffusion MRI space to the common space. Diffusion tensor fitting was performed using the b = 750 s/mm^2^ volumes as implemented in FSL’s dtifit, and the resulting FA images were warped to the common space.

### 2.3 Human traumatic brain injury data collection

#### 2.3.1 Magnetic resonance imaging data collection

All participants were scanned at the earliest availability of the hospital’s 3T MRI scanner (Prisma, Siemens Healthcare, Germany) after they were determined by the treating physician to be sufficiently clinically stable, using a 32-channel head array coil. The median time post-injury interval was 8 days, and the interquartile range was 3–13 days. Due to COVID restrictions, imaging was delayed to 114 days for one patient and 213 days for another patient. T2-weighted (T2w) fluid-attenuated inversion recovery (FLAIR) was performed with the following parameters: TE = 81 ms, TR = 9000 ms, FA = 150 degrees, and effective resolution = 0.69 × 0.69 × 3.3 mm. A structural T1-weighted (T1w) magnetisation prepared rapid gradient-echo (T1w-MPRAGE) sequence was performed with the following parameters: TE = 2.26 ms, TR = 1900 ms, FA = 9 degrees, effective resolution = 1 mm^3^ isotropic. The diffusion MRI image was acquired with the following parameters: TE = 84 ms, TR = 4700 ms, FA = 90 degrees, and effective resolution = 2 mm^3^ isotropic, 60 diffusion-weighted directions at b value = 3000 s/mm^2^ and 12 b0 volumes.

#### 2.3.2 Blood sampling, processing, and quantitation of immune cytokine concentrations

On the day of the MRI scan, blood samples were obtained from the TBI participants using EDTA collection tubes. A 10 ml sample of whole blood was collected and centrifuged at 1600 g for 10 minutes. The resulting serum was then carefully removed and stored at -80 °C until the time of analysis. The multiplexed immunoassay procedure utilized the Bio-Plex Pro Human Cytokine 27-plex Assay (Catalogue number: M500KCAF0Y) following the provided guidelines without any alterations. In summary, the frozen samples were defrosted, mixed using a vortex, and subsequently centrifuged at 1000 g for 2 minutes at 4 °C. As per the manufacturer’s instructions, the samples were diluted at a ratio of 1:4 with the sample diluent from the kit, whereupon the diluted samples were passed through 0.22 µm high recovery Durapore (PVDF) centrifuge filters with rotation at 14,000 g for 15 minutes at 4 °C.

For the analysis, both standards and samples were processed using a 96-well plate and a robotic liquid handling workstation (epMotion 5075; Eppendorf). Subsequent washing of the plate was carried out using a Bio-Plex Pro II magnetic plate washer (Bio-Rad), and the readings were taken using the Bio-Plex Systems 200. Throughout the incubation process at 25 °C, the assay plates were gently agitated at 850 rpm under subdued light. The Bio-Plex Manager v6.0 software was employed to analyse the samples and generate standard curves. In instances where the measured concentration of a specific analyte was below the detectable range of the standard curves, the value was recorded as zero. The following biomarkers were quantified: interleukins (IL) IL-1β, IL-4, IL-6, IL-7, IL-8, IL-9, IL-17, and interleukin 1 receptor antagonist (IL-1ra), as well as Eotaxin, basic fibroblast growth factor (bFGF), granulocyte colony-stimulating factor (G-CSF), interferon gamma (IFN-γ), chemokines (C-C motif) ligands 2, 3, 4, and 5 (CCL2, CCL3, CCL4, and CCL5), platelet-derived growth factor-BB (PDGF-BB), and tumour necrosis factor alpha (TNF-α).

#### 2.3.3 Magnetic resonance imaging data processing

Each participant’s T2w-FLAIR image was rigidly registered to their T1w-MPRAGE image, which was corrected for bias field inhomogeneity and segmented into grey matter, white matter, cerebrospinal fluid (CSF), skull, and head tissue probability maps using SPM12’s Segment tool. The TBI lesion area was identified as hyper-intense regions on T2w-FLAIR and hypo-intense or iso-intense regions with normal grey matter on T1w-MPRAGE using the region competition snakes segmentation (37) implemented in ITK-SNAP (v.3.8) with the following procedure: the co-registered and bias field-corrected T1w-MPRAGE and T2w-FLAIR images of each participant were loaded into ITK-SNAP and a multi-modal tissue class classifier pre-segmentation step was employed to produce the preliminary segmentation probabilistic map. Representative examples of the TBI lesion (as defined above), normal grey matter, normal white matter, and the cerebrospinal fluid were manually identified for each participant. These example regions served as tissue class classifiers for the semi-automatic classification pre-segmentation step, which generate tissue class probabilistic maps. The tissue class probabilistic map for the lesion area were inspected (by XVT) to ensure that it reasonably delineated the TBI lesion areas; inevitably, the lesion probabilistic maps included non-lesion and non-brain areas, but over the positively identified lesion areas, we confirmed that the lesion probabilistic maps did not include the adjacent normal-appearing brain areas. Once we were satisfied with the probabilistic maps, initiation bubbles were seeded over the suspected lesion areas and iterative snake evolution was performed to grow the initiation bubbles to cover the lesion areas. Here, the curvature parameter was set to 0.001.

The grey matter, white matter, and CSF tissue probabilistic maps from SPM12’s segmentation of the T1w-MPRAGE image and the segmented TBI lesion map created as above were combined and binarized to generate a whole brain mask including the lesion area included (typical skull-stripping algorithms like FSL’s BET and SPM12’s Segment often miss brain lesion areas). This whole brain mask was used to generate a bias field inhomogeneity-corrected and skull-stripped T1w-MPRAGE structural image. The generated whole brain mask image was subtracted by the TBI lesion map to generate a normal appearing brain (NAB) mask, which include the normal appearing grey matter, white matter, and the CSF.

The b0 value images were extracted and merged into a 4D image, which was then motion-corrected using FSL’s eddy_correct and averaged to generate a b0 spatial representation of diffusion MRI data. This b0 spatial representation image was N4ITK bias field-corrected (38), and then skull-stripped using FSL’s BET. The resultant spatial representation image was affinely registered to the inhomogeneity-corrected and skull-stripped T1w-MPRAGE structural image. Diffusion MRI data were fitted to the diffusion tensor model using FSL’s dtifit.

All participants’ T1w-MPRAGE images were registered to the OASIS template of the MICCAI 2012 workshop on multi-atlas labelling (39) using the CCFM approach (34), with the NAB mask serving as the cost function mask for the individual T1w-MPRAGE image and the OASIS template mask as the cost function mask for the OASIS template. The deformation model was the Syn-CC model (35) as implemented in ANTS (v.2.3.4) (36). The warping field obtained from this step was combined with the transformation matrix obtained from the diffusion MRI-to-T1w-MPRAGE registration step to warp the human diffusion MRI metrics to a common space.

### 2.4 Statistical analysis

Statistical analysis was performed in GraphPad Prism (v.9.5.1, GraphPad Software, Inc., San Diego, California, USA). Normality of the data was tested using the Shapiro-Wilk test (40, 41).

#### 2.4.1 Animal data statistical analysis

Unpaired Mann-Whitney tests were performed to compare plasma levels of immunological biomarkers in the sham and TBI groups at different timepoints, with correction for multiple comparisons using the two-stage linear step-up procedure of Benjamini, Krieger, and Yekutieli for false discovery rate correction (42). FDR threshold was set at the desired FDR (Q) < 0.1.

Data reduction for TBI animals’ plasma immunological markers at each timepoint was accomplished using principal component analysis (PCA) in Prism (v.9.5.1). The plasma concentrations of each biomarker across all the TBI animals at each timepoint (days 1, 3, 7, or 60 post-TBI) were standardised and centred so that the mean was zero and standard deviation was unity for each biomarker across the TBI animals. We performed PCA on these standardised and centred plasma concentrations at each timepoint and selected a maximum of three principal components (PCs) for each PCA to minimise over-fitting. The summary results for the extracted PCs for the immunological biomarker panel at each timepoint are presented in **Tables 1A–D**. We next used the extracted PC scores to perform voxel-wise multiple linear regression (MLR) (this procedure is also known as Principal Component Regression [PCR] (43)) predicting the outcome of DTI metrics at day 60 post-injury using each TBI animal’s plasma immunological biomarker panel at each timepoint. The voxel-wise MLR was performed using permutation inference for the general linear model (44) in FSL’s *randomise* (45), with the number of permutations set to 10,000 or exhaustive, whichever was smaller. The resulting statistical maps were corrected for multiple comparisons with mass-based FSL’s threshold-free cluster enhancement (TFCE) (46) and thresholded at P value < 0.05 (two-tailed).

**Table 1:** Summary of the principal components (PCs) extracted from the plasma immunological panels of rat controlled cortical impact (CCI) model of traumatic brain injury (TBI) days 1 (a), 3 (b), 7 (c), and 60 (d) post-TBI, and of (e) human TBI patients with a median time post-injury of 8 days.

(a) Rat day 1 post-TBI immunological marker levels			
PC summary	PC1	PC2	PC3
Eigenvalue	16.5	4.2	1.4
Proportion of variance (%)	71.6	18.1	6.1
Cumulative proportion of variance (%)	71.6	89.7	95.8
(b) Rat day 3 post-TBI immunological marker levels			
PC summary	PC1	PC2	PC3
Eigenvalue	13.9	5.9	1.8
Proportion of variance (%)	60.6	25.7	7.8
Cumulative proportion of variance (%)	60.6	86.3	94.2
(c) Rat day 7 post-TBI immunological marker levels			
PC summary	PC1	PC2	PC3
Eigenvalue	9.1	6.0	4.2
Proportion of variance (%)	39.5	26.1	18.2
Cumulative proportion of variance (%)	39.5	65.5	83.7
(d) Rat day 60 post-TBI immunological marker levels			
PC summary	PC1	PC2	PC3
Eigenvalue	10.7	5.6	3.4
Proportion of variance (%)	46.4	24.3	14.6
Cumulative proportion of variance (%)	46.4	70.6	85.2
(e) Human clinical post-TBI immunological marker levels			
PC summary	PC1	PC2	PC3
Eigenvalue	5.7	5.2	4.1
Proportion of variance (%)	26.9	24.7	19.7
Cumulative proportion of variance (%)	26.9	51.7	71.4

**Figure 1:**
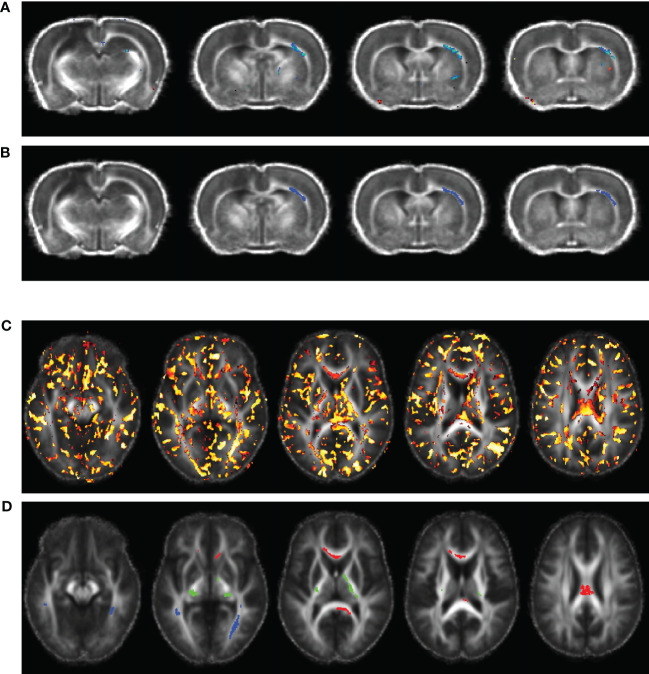
Voxel-wise multiple linear regression results and the identified regions-of-interest (ROIs) correlating animal **(A, B)** and human **(C, D)** fractional anisotropy (FA) and the principal components (PCs) extracted from the corresponding plasma immunological biomarkers. The images were overlaid on the averaged registered FA maps. **(A)** Statistical map of multiple linear regression analysis correlating the FA map with the PCs extracted from plasma immunological biomarkers panel at day 60 post-injury. **(B)** ROI selected for the rat data in the right external capsule (blue, ec), ipsilateral to the injury. **(C)** Statistical map of multiple linear regression analysis correlating the TBI patients’ FA maps with the PCs extracted from the corresponding plasma immunological biomarkers, at a median of eight days post injury. **(D)** ROIs selected for the human data in the corpus callosum (red, cc), corticospinal tract (green, cst), and longitudinal fasciculus (blue, lst).

The voxel-wise regression analysis identified only one region-of-interest (ROI), namely the external capsule white matter tract contralateral to the injured hemisphere, in which FA correlated the animals’ PCA-reduced plasma immunological biomarker panel at day 60 post-TBI, as displayed in **Figures 1A, B**. FA values were extracted from the identified ROI and used to perform a PCR (43) to correlate the FA value from the ROI with the plasma immunological biomarker panel results, with up to 3 PCs, as above. The PCR extracted the PC scores from the PCA step and conducted MLR correlating the variation in the FA of the ROI with the 3 PC scores and the resulting regression coefficients were back-projected to the original immunological biomarkers; this step served to enable a more intuitive understanding the results quantifying the relationship between FA variation and the variation of each immunological plasma biomarker. The validity of the MLR model was tested through a Shapiro-Wilk’s normality test (40) of the residuals.

#### 2.4.2 Human data statistical analysis

Unpaired Mann-Whitney tests comparing the plasma levels of immunological biomarkers were performed to compare TBI and control participants, with significance set at P value < 0.05. As with the animal plasma immunological biomarker panel, we performed data reduction of the human biomarker panel by PCA, with a maximum of three PCs. A summary the extracted PCs is presented in **Table 1E**. The extracted PC scores were then used to perform voxel-wise MLR for correlating the DTI metrics in TBI patients against their PC scores which explained most of the variations in the plasma immunological biomarker panel. Implementation of the voxel-wise MLR analysis in the human data was much as for the animal data, and similarly identified regions in the patients’ white matter in which FA correlated with the biomarker panel. We identified three ROIs for further analysis: corpus callosum (cc), longitudinal fasciculus (lf), and the corticospinal tract (cst), as shown in **Figures 1C, D**. FA values were extracted from these ROIs were PCR analysis for correlation with the data reduced plasma immunological biomarker panel. The analysis steps were like those performed on the animal data as presented above.

## 3 Results

We have presented a comparison of the plasma immunological biomarkers between sham and TBI groups in these rats in an earlier manuscript (30). In brief, IL-1β, IL-7, GM-CSF, and CCL3 were elevated in the TBI group compared to the sham group between day 3 and day 60 post-injury. IL-2, IL-4, IL-5, IL-6, IL-13, IL-17α, IL-19, G-CSF, and IFN-γ extremely elevated in several TBI animals at day 1–7 post-TBI though statistical analysis did not show significant group differences at all timepoint post-injury and the animals with extreme day 1–7 values apparently normalised at day 60. CCL2, CCL20, TNF-α, IL-18, and VEGF never differed significantly between the TBI and sham group. IL-1α, IL-12p70, and M-CSF were decreased in the TBI group compared to sham group only at day 1 post-injury and there was no difference at day 3, 7, or 60.

**Figure 2:**
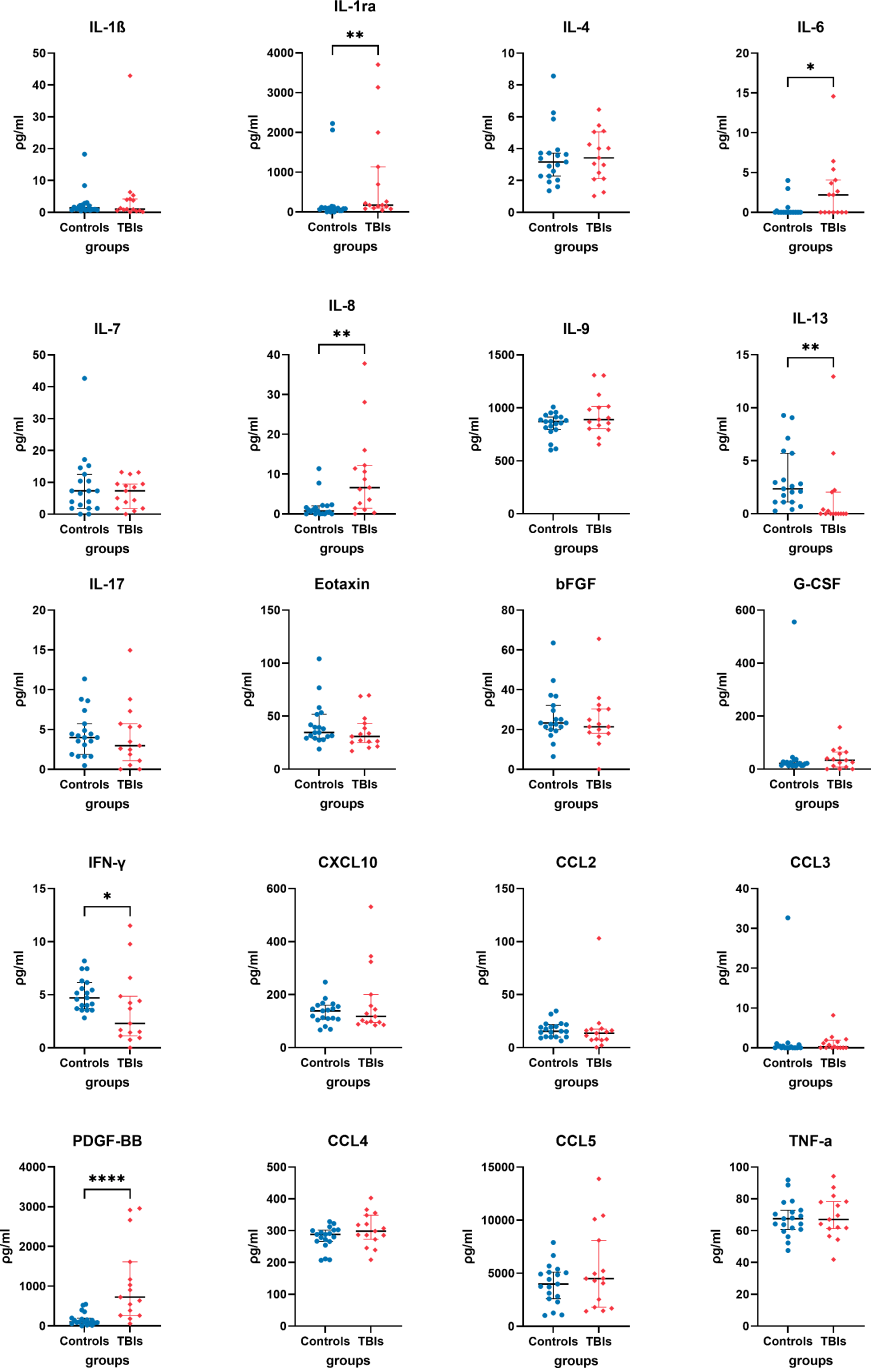
Quantified plasma concentrations of immunological biomarkers from traumatic brain injury and control group of interleukin (IL)-1β, interleukin 1 receptor antagonist (IL-1ra), IL-4, IL-6, IL-7, IL-8, IL-13, IL-17, Eotaxin, basic fibroblast growth factor (bFGF), granulocyte colony-stimulating factor (G-CSF), interferon gamma (IFN-γ), granulocyte colony-stimulating factor (G-CSF), C-X-C motif chemokine ligand 10 (CXCL10), chemokine (C-C motif) ligands 2 (CCL2), CCL3, platelet-derived growth factor-BB (PDGF-BB), CCL4, CCL5, tumour necrosis factor alpha (TNF-α). Mann-Whitney rank test, * P value < 0.05, ** P value < 0.01, **** P value < 0.0001. Bars and whiskers presented the median and inter-quartile range.

Analysis of human TBI and control plasma immunological biomarker panels showed that IL-1ra, IL-6, IL-8, and PDGF-BB were significantly elevated and IL-13, IFN-γ significantly lower in the TBI compared to control participants (**Figure 2**). IL-1β, IL-4, IL-7, IL-9, IL-17, Eotaxin, bFGF, G-CSF, CXCL10, CCL2, CCL3, CCL4, CCL5, and TNF-α showed insignificant differences between TBI and control participants.

**Figure 3:**
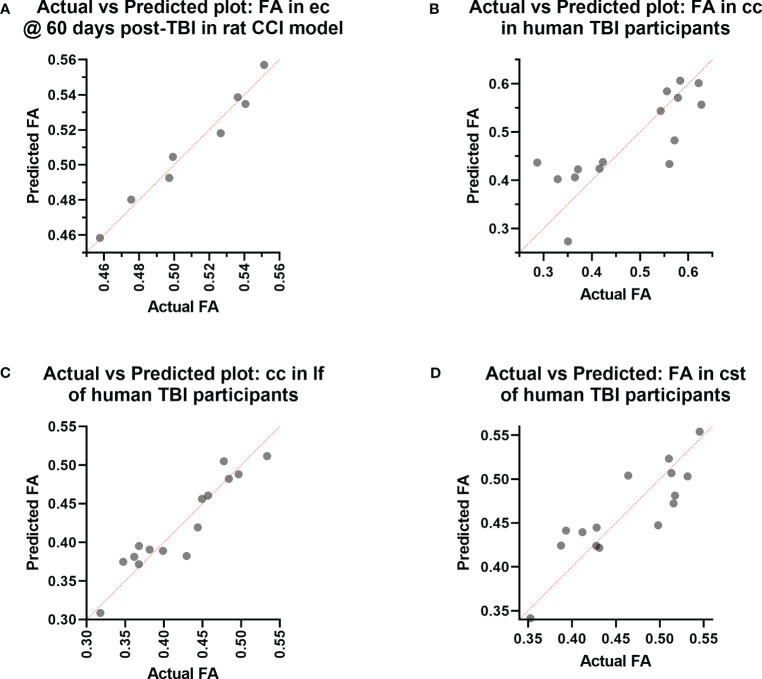
Actual vs. Predicted value results of the Principal Component Regression correlating the **(A)** fractional anisotropy (FA) in the external capsule (ec) region-of-interest (ROI) at day 60 post-injury with the plasma immunological panel at day 60 post-injury in the rat controlled cortical impact (CCI) model of traumatic brain injury (TBI), and the **(B)** FA in the corpus callosum (cc) ROI, **(C)** FA in the longitudinal fasciculus (lf) ROI, and **(D)** FA in the corticospinal tract (cst) ROI with the plasma immunological panel in clinical traumatic brain injury (TBI) patients at a median of eight days post-injury.

In the experimental animals, voxel-wise MLR analysis showed that FA in the contralateral (to the injured hemisphere) ec at day 60 post-TBI correlated with the day 60 post-TBI plasma immunological biomarker panel (**Figure 1A**). ROI-based analysis showed that the FA in the rat ec ROI correlated positively with the day 60 plasma levels of IL-1α, IL-1β, IL-2, IL-4, IL-5, IL-6, IL-7, IL-10, IL-12p70, IL-17α, IL-18, G-CSF, GM-CSF, IFN-γ, CXCL1, CCL2, CCL3, VEGF, and TNF-α. The regression equation was significant (**Table 2**, F [3, 4] = 46.6, P value = 0.0014 and **Figure 3A**) with an adjusted R^2^ of 0.951. The residuals passed the normality test (Shapiro-Wilk test, P value = 0.212), and the MLR model was thus considered valid.

Table 2Results of principal component regression (PCR) correlating the fractional anisotropy (FA) in the external capsule (ec) region-of-interest (ROI) at day 60 post-injury with the plasma immunological panel at day 60 post-injury in the rat controlled cortical impact (CCI) model of traumatic brain injury (TBI).

**Table:** 

Analysis of Variance	SS	DF	MS	F (DFn, DFd)	P value
Regression	7.59E-03	3	2.53E-03	F (3, 4) = 46.6	**P=0.0014**
Residual	2.17E-04	4	5.43E-05		
Total	7.81E-03	7			

Dependent variable: FA in the external capsule at 60 days post-TBI in rat CCI model.

Predictor variable: plasma immunological panel at 60 days post-TBI in rat CCI model. Bolded values indicate P < 0.05.

**Table:** 

Parameter estimates	Variable	Estimate	95% CI (asymptotic)			|t|	P value
β0	Intercept	4.07E-01	3.73E-01	to	4.41E-01	33.5	**<0.0001**
β1	IL-1β	1.35E-05	7.40E-06	to	1.96E-05	6.14	**0.0036**
β2	IL-1α	2.84E-05	9.18E-06	to	4.77E-05	4.1	**0.0148**
β3	IL-2	1.61E-06	6.80E-07	to	2.54E-06	4.81	**0.0086**
β4	IL-4	3.08E-05	1.64E-05	to	4.51E-05	5.96	**0.0040**
β5	IL-5	3.01E-05	1.39E-05	to	4.63E-05	5.16	**0.0067**
β6	IL-6	2.76E-05	1.73E-05	to	3.78E-05	7.45	**0.0017**
β7	IL-7	1.63E-05	9.31E-06	to	2.33E-05	6.46	**0.0030**
β8	IL-10	5.08E-05	3.18E-05	to	6.97E-05	7.44	**0.0017**
β9	IL-12p70	2.31E-05	1.52E-05	to	3.09E-05	8.16	**0.0012**
β10	IL-13	9.29E-06	-2.31E-06	to	2.09E-05	2.22	0.0903
β11	IL-17α	3.06E-04	1.88E-04	to	4.24E-04	7.2	**0.0020**
β12	IL-18	2.96E-07	3.13E-09	to	5.88E-07	2.81	**0.0485**
β13	G-CSF	7.54E-04	3.88E-04	to	1.12E-03	5.72	**0.0046**
β14	M-CSF	-6.56E-05	-2.56E-04	to	1.24E-04	0.959	0.3920
β15	GM-CSF	1.56E-05	8.06E-06	to	2.32E-05	5.73	**0.0046**
β16	IFN-γ	2.81E-05	1.81E-05	to	3.81E-05	7.78	**0.0015**
β17	CXCL1	2.57E-05	1.41E-05	to	3.74E-05	6.12	**0.0036**
β18	CCL2	1.22E-05	6.83E-06	to	1.76E-05	6.3	**0.0032**
β19	CCL3	8.66E-05	5.12E-05	to	1.22E-04	6.79	**0.0025**
β20	CCL5	2.77E-05	-9.33E-06	to	6.48E-05	2.08	0.1064
β21	CCL20	4.96E-05	-2.11E-04	to	3.10E-04	0.528	0.6255
β22	VEGF	1.94E-05	2.60E-06	to	3.62E-05	3.21	**0.0327**
β23	TNF-α	1.48E-05	9.52E-06	to	2.01E-05	7.8	**0.0015**

Adjusted R squared = 0.951.

**Table:** 

Normality of Residuals	Statistics	P value	Passed normality test (alpha=0.05)?
Shapiro-Wilk (W)	0.8854	0.2121	Yes

Voxel-wise MLR showed that the FA in various human white matter regions correlated significantly with the plasma immunological biomarker panel (**Figure 1C**). FA specifically in the cc ROI had a significant positive correlation with IL-6, IL-8, IL-9, G-CSF, CXCL10, CCL3, PDGF-BB, CCL4, CCL5, and a negative correlation with IL-13 and CCL2. The regression equation was significant (**Table 3**, F [3, 11] = 6.70, P = 0.0078 and **Figure 3B**), with an adjusted R^2^ of 0.550. The residuals passed the normality test (Shapiro-Wilk test, P value = 0.97) and the MLR model was thus considered valid. FA in the lf ROI correlated positively with IL-1β, IL-1ra, IL-6, IL-8, IL-9, bFGF, G-CSF, CXCL10, CCL3, PDGF-BB, CCL4, CCL5, TNF-α and negatively with IL-4, IL-7, IL-13, Eotaxin, and CCL2. The regression equation was significant (**Table 4**, F [3, 11] = 28.4, P value < 0.0001 and **Figure 3C**) with an adjusted R^2^ of 0.854. The residuals passed the normality test (Shapiro-Wilk test, P value = 0.4382) and the MLR model was thus considered valid. FA in the cst correlated positively with the plasma levels of IL-1ra, IL-6, IL-8, IL-9, G-CSF, CXCL10, CCL3, PDGF-BB, CCL4, CCL5, and negatively with IL-4, IL-7, IL-13, Eotaxin, and CCL2. The regression equation was significant (**Table 5**, F (3, 11) = 10.6, P value = 0.0014 and **Figure 3D**), with an adjusted R^2^ of 0.672. The residuals passed normality test (Shapiro-Wilk test, P value = 0.75) and the MLR model was thus considered valid.

Table 3Results of principal component regression (PCR) correlating the fractional anisotropy (FA) in the corpus callosum (cc) region-of-interest (ROI) with the plasma immunological panel in clinical traumatic brain injury (TBI) patients at a median of 8 days post injury.

**Table:** 

Analysis of Variance	SS	DF	MS	F (DFn, DFd)	P value	
Regression	1.26E-01	3	4.21E-02	F (3, 11) = 6.70	**P=0.0078**
Residual	6.91E-02	11	6.28E-03			
Total	1.95E-01	14				

Dependent variable: FA in the corpus callosum ROI of post-TBI in participants.

Predictor variable: plasma immunological panel panel post-TBI in participants.Bolded values indicate P < 0.05.

**Table:** 

Parameter estimates	Variable	Estimate	95% CI (asymptotic)			|t|	P value
β0	Intercept	3.02E-01	1.14E-01	to	4.91E-01	3.53	**0.0047**
β1	IL-1β	2.37E-04	-5.92E-04	to	1.07E-03	0.629	0.5424
β2	IL-1ra	5.59E-06	-1.23E-06	to	1.24E-05	1.8	0.0988
β3	IL-4	-3.37E-03	-8.69E-03	to	1.94E-03	1.4	0.1901
β4	IL-6	2.54E-03	1.14E-03	to	3.94E-03	3.99	**0.0021**
β5	IL-7	-9.53E-04	-2.17E-03	to	2.60E-04	1.73	0.1118
β6	IL-8	1.12E-03	3.60E-04	to	1.89E-03	3.24	**0.0079**
β7	IL-9	4.80E-05	5.14E-06	to	9.09E-05	2.47	**0.0314**
β8	IL-13	-3.84E-03	-6.24E-03	to	-1.44E-03	3.52	**0.0048**
β9	IL-17	1.29E-04	-2.42E-03	to	2.68E-03	0.112	0.9132
β10	Eotaxin	-3.57E-04	-8.19E-04	to	1.05E-04	1.7	0.1169
β11	bFGF	4.16E-04	-2.39E-04	to	1.07E-03	1.4	0.1897
β12	G-CSF	2.27E-04	7.68E-05	to	3.77E-04	3.33	**0.0068**
β13	IFN-γ	-2.15E-03	-5.25E-03	to	9.46E-04	1.53	0.1546
β14	CXCL10	7.62E-05	2.53E-05	to	1.27E-04	3.3	0.0071
β15	CCL2	-5.02E-04	-8.25E-04	to	-1.80E-04	3.43	**0.0056**
β16	CCL3	5.76E-03	2.34E-03	to	9.19E-03	3.7	**0.0035**
β17	PDGF-BB	1.44E-05	6.27E-06	to	2.26E-05	3.89	**0.0025**
β18	CCL4	2.01E-04	5.71E-05	to	3.44E-04	3.08	**0.0106**
β19	CCL5	2.90E-06	7.53E-07	to	5.05E-06	2.97	**0.0127**
β20	TNF-α	4.59E-04	-1.20E-04	to	1.04E-03	1.75	0.1087

Adjusted R squared = 0.550.

**Table:** 

Normality of Residuals	Statistics	P value	Passed normality test (alpha=0.05)?
Shapiro-Wilk (W)	0.9696	0.8528	Yes

Table 4Results of the principal component regression (PCR) correlating the fractional anisotropy (FA) in the longitudinal fasciculus (lf) region-of-interest (ROI) with the plasma immunological panel in clinical traumatic brain injury (TBI) patients at a median of 8 days post injury.

**Table:** 

Analysis of Variance	SS	DF	MS	F (DFn, DFd)	P value
Regression	4.93E-02	3	1.64E-02	F (3, 11) = 2z8.4	**P<0.0001**
Residual	6.36E-03	11	5.78E-04		
Total	5.57E-02	14			

Dependent variable: FA in the longitudinal fasciculus ROI of post-TBI in participants.

Predictor variable: cytokine panel post-TBI in participants.Bolded values indicate P < 0.05.

**Table:** 

Parameter estimates	Variable	Estimate	95% CI (asymptotic)			|t|	P value
β0	Intercept	3.05E-01	2.47E-01	to	3.62E-01	11.7	**<0.0001**
β1	IL-1β	3.47E-04	9.60E-05	to	5.99E-04	3.04	**0.0112**
β2	IL-1ra	4.78E-06	2.71E-06	to	6.85E-06	5.09	**0.0004**
β3	IL-4	-1.65E-03	-3.26E-03	to	-3.27E-05	2.25	**0.0462**
β4	IL-6	1.52E-03	1.10E-03	to	1.95E-03	7.88	**<0.0001**
β5	IL-7	-6.45E-04	-1.01E-03	to	-2.77E-04	3.86	**0.0027**
β6	IL-8	8.15E-04	5.83E-04	to	1.05E-03	7.75	**<0.0001**
β7	IL-9	2.52E-05	1.22E-05	to	3.82E-05	4.27	**0.0013**
β8	IL-13	-2.01E-03	-2.74E-03	to	-1.28E-03	6.07	**<0.0001**
β9	IL-17	6.13E-04	-1.61E-04	to	1.39E-03	1.74	0.1089
β10	Eotaxin	-1.96E-04	-3.36E-04	to	-5.54E-05	3.07	**0.0106**
β11	bFGF	3.90E-04	1.91E-04	to	5.88E-04	4.32	**0.0012**
β12	G-CSF	1.60E-04	1.14E-04	to	2.06E-04	7.73	**<0.0001**
β13	IFN-γ	-6.02E-04	-1.54E-03	to	3.37E-04	1.41	0.1857
β14	CXCL10	3.94E-05	2.40E-05	to	5.49E-05	5.63	**0.0002**
β15	CCL2	-2.71E-04	-3.69E-04	to	-1.73E-04	6.1	**<0.0001**
β16	CCL3	3.75E-03	2.71E-03	to	4.79E-03	7.95	**<0.0001**
β17	PDGF-BB	8.62E-06	6.14E-06	to	1.11E-05	7.65	**<0.0001**
β18	CCL4	1.14E-04	7.02E-05	to	1.57E-04	5.74	**0.0001**
β19	CCL5	1.63E-06	9.75E-07	to	2.28E-06	5.49	**0.0002**
β20	TNF-α	3.04E-04	1.29E-04	to	4.80E-04	3.82	**0.0028**

Adjusted R squared = 0.854.

**Table:** 

Normality of Residuals	Statistics	P value	Passed normality test (alpha=0.05)?
Shapiro-Wilk (W)	0.9442	0.4382	Yes

Table 5Results of principal component regression (PCR) correlating the fractional anisotropy (FA) in the corticospinal tract (cst) region-of-interest (ROI) with the plasma immunological panel in clinical traumatic brain injury (TBI) patients at a median of eight days post-injury.

**Table:** 

Analysis of Variance	SS	DF	MS	F (DFn, DFd)	P value	
Regression	3.86E-02	3	1.29E-02	F (3, 11) = 10.6	**P=0.0014**
Residual	1.34E-02	11	1.22E-03			
Total	5.21E-02	14				

Dependent variable: FA in the corticospinal tract ROI of post-TBI in participants.

Predictor variable: cytokine panel post-TBI in participants.Bolded values indicate P < 0.05.

**Table:** 

Parameter estimates	Variable	Estimate	95% CI (asymptotic)			|t|	P value
β0	Intercept	4.02E-01	3.19E-01	to	4.85E-01	10.7	**<0.0001**
β1	IL-1β	9.12E-05	-2.74E-04	to	4.56E-04	0.55	0.5935
β2	IL-1ra	3.46E-06	4.54E-07	to	6.47E-06	2.53	**0.0278**
β3	IL-4	-2.93E-03	-5.27E-03	to	-5.91E-04	2.76	**0.0187**
β4	IL-6	1.52E-03	9.02E-04	to	2.14E-03	5.42	**0.0002**
β5	IL-7	-7.45E-04	-1.28E-03	to	-2.11E-04	3.07	**0.0107**
β6	IL-8	6.15E-04	2.79E-04	to	9.52E-04	4.03	**0.0020**
β7	IL-9	1.98E-05	9.60E-07	to	3.87E-05	2.31	**0.0411**
β8	IL-13	-2.25E-03	-3.31E-03	to	-1.19E-03	4.68	**0.0007**
β9	IL-17	-3.08E-04	-1.43E-03	to	8.16E-04	0.603	0.5586
β10	Eotaxin	-2.88E-04	-4.91E-04	to	-8.47E-05	3.12	**0.0098**
β11	bFGF	1.40E-04	-1.48E-04	to	4.28E-04	1.07	0.3084
β12	G-CSF	1.31E-04	6.53E-05	to	1.98E-04	4.38	**0.0011**
β13	IFN-γ	-1.33E-03	-2.69E-03	to	3.34E-05	2.15	0.0549
β14	CXCL10	3.86E-05	1.62E-05	to	6.10E-05	3.79	**0.0030**
β15	CCL2	-3.10E-04	-4.52E-04	to	-1.68E-04	4.8	**0.0006**
β16	CCL3	3.46E-03	1.95E-03	to	4.97E-03	5.05	**0.0004**
β17	PDGF-BB	7.01E-06	3.41E-06	to	1.06E-05	4.29	**0.0013**
β18	CCL4	8.91E-05	2.58E-05	to	1.52E-04	3.1	**0.0101**
β19	CCL5	1.28E-06	3.28E-07	to	2.22E-06	2.96	**0.0129**
β20	TNF-α	1.31E-04	-1.24E-04	to	3.85E-04	1.13	0.2821

Adjusted R squared = 0.672.

**Table:** 

Normality of Residuals	Statistics	P value	Passed normality test (alpha=0.05)?
Shapiro-Wilk (W)	0.9635	0.7524	Yes

## 4 Discussion

In the rat model of TBI and likewise in the group of TBI patients, there was generally agreement in the correlations between the biomarkers and diffusion MRI indicators of white matter integrity, thus concurring that the observed changes in plasma biomarkers were associated with a greater degree of white matter integrity, albeit with imperfect agreement of the direction of correlations between rat TBI and human results. In the rat CCI model, plasma immunological biomarker levels at day 60 post-injury were generally associated with higher FA in the ec ROI, which general indicates higher white matter integrity in the ec at day 60 post-injury (decreased FA is generally associated with poorer white matter integrity (47)). Also, the CCI model rats at day 60 generally had significantly elevated levels of the same biomarkers or had shown increased levels at day 3-7 that had normalised at day 60. Mean plasma levels of IL-1ra, IL-6, IL-8, and PDGF-BB were significantly higher in the TBI participants compared to control samples mostly between day 3 and day 13 post-injury; these biomarkers correlated positively with FA in several white matter ROIs. Conversely, IL-13 and IFN-γ levels were lower in TBI participants as compared to control samples, and these same biomarkers in the patients correlated negatively with FA.

While the direction of changes in the immunological biomarker and of the association between these biomarkers and white matter FA did not match in CCI rats and clinical TBI cases, there was a general agreement that levels of a short list of biomarkers were associated with preserved white matter integrity. Cross-species results regarding the direction of correlation were consistent for IL-1β, IL-6, G-CSF, CCL3, CCL5, and TNF-α, but differed for IL-4, IL-7, IL-13, and CCL2. The generally positively correlating relationships of biomarker levels and white matter integrity in the TBI model rats occurred with along with elevations in most of those same biomarkers post-TBI. We see a similar phenomenon in the TBI participants, whereby certain biomarkers were elevated, and correlated positively with FA in the white matter ROIs. However, the positive correlation between IL-13 levels with FA in the rat model stands in contrast to the negative correlation seen in human TBI patients; whereas relative to control samples, the human TBI participants showed a significantly reduced plasma IL-13 level at a median of 8 days post injury, a few of the TBI rats had extremely high IL-13 levels in the first week post-injury. IL-13 administration in a mouse model of open-head moderate-severe TBI reduced neuronal loss and preserved white matter integrity (48). IL-4 and IL-7 had differing trends in correlation with FA; although IL-4 and IL-7 concentrations in the human TBI patients’ samples did not differ from control values, several rats had extremely high levels of IL-4 and IL-7 in the first week post-injury (as seen likewise in the same rats for IL-13) compared to controls. The interpretation of the extremely high levels in a few animals is uncertain, since those animals did not have conspicuously larger TBI lesions, greater tissue loss, nor notable functional deficits (30). CCL2 levels did not significantly differ in the human or rat TBI samples as compared to control results. We conclude that, while the specific direction of change and effects of the immunological biomarkers can differ between animal and human TBI, the observed changes are generally associated with preserved white matter integrity in the face of the injury.

Certain biomarkers were significantly elevated in the present human TBI participants to an extent correlating significantly to their white matter integrity, with an R^2^ of the MLR model ranging from 0.550 to 0.854. Among these, we previously found in a similar clinical TBI cohort that plasma IL-1ra correlated with higher BBB integrity in the primary TBI brain lesion (49). As an endogenous inhibitor of the pro-inflammatory IL-1 (50), IL-1ra is an important anti-inflammatory protein in certain autoimmune diseases, including rheumatoid arthritis, colitis, and granulomatous pulmonary disease (51), and has garnered interest as a therapeutic intervention in CNS disorders with an inflammatory component (50). We also found elevation of plasma IL-6 and IL-8 in the present TBI participants, to an extent correlating with white matter FA, which could suggest a protective effect. On the other hand, higher CSF levels of IL-6, IL-8, and other markers including IFN-γ, IL-1β, and TNF-α were associated with unfavourable outcomes in human TBI patients (52). Furthermore, higher plasma levels of IL-6 (53–56) and IL-8 (53, 56, 57) were associated with post-TBI mortality and respiratory distress (55, 56) in TBI patients. That higher levels of these biomarkers were associated with preserved FA in the present group of TBI patients may reflect our recruitment procedure; the patients were scanned and had blood drawn only when they were deemed sufficiently stable by their treating physicians after emergency treatment and/or intensive care, i.e., a median of 8 days after the injury.

We also found elevated plasma levels of PDGF-BB, which showed a positive correlation with white matter FA. Intravenous administration of PDGF-BB inhibited endoplasmic reticulum and autophagy stress and promoted neurological recovery in a mouse model of open-head TBI (58). In another rodent model, TBI interfered with PDGF signalling in a manner contributing to BBB dysfunction (59). Thus, we see general agreement that PDGF-BB may mediate recovery from TBI.

The biomarkers that were elevated in TBI model rats and in the patients are generally known to be associated with negative outcomes post-TBI. Nonetheless, we report in this study that elevations in several cytokines were associated with higher FA in the white matter both in human participants and the experimental animals. This discrepancy may bear some relation to acutely life-threatening outcomes such as multiple organ failure and respiratory distress in the previous clinical studies. However, all our participants were sufficiently stable once they were deemed ready for MRI, and hence had survived their injury. Since neuroinflammation is neither strictly detrimental nor beneficial (9), present results elude simple interpretation. Furthermore, biomarkers generally show dynamic changes in the aftermath of the injury. For example, our earlier analyses of the rat immunological panel results indicated a narrow time window of approximately 7 days post injury (30) in which there was an association with the trans-hemispheric cortical map transfer process that occurs after TBI (60–64); we argued that the trans-hemispheric cortical map transfer could be a pathological process (30). Specifically, among the biomarkers in this study that we found to have consistent correlation direction between animals and humans (IL-1β, IL-6, G-CSF, CCL3, CCL5, and TNF-α) or differing correlation direction (IL-4, IL-7, IL-13, and CCL2), CCL3 and CCL5 were both significantly increased in TBI animals at day 7 post-injury and associated with a lesser extent of cortical map transfer (and potentially lesser pathology), IL-1β and IL-7 were significantly increased but no apparent effect on the cortical map transfer, TNF-α and CCL2 were not significantly different between TBI and sham animals, and IL-6, G-CSF, IL-4, and IL-13 were extremely elevated in a few animals and associated with a greater extent of cortical map transfer (and potentially pathology) (30). CCL3 is an anti-inflammatory chemokine which had mRNA upregulation in the peripheral blood in a rat model of lateral fluid percussion injury (65). CCL5 protected hippocampal memory function in a mouse drop weight model of mild TBI (66).

We have also found that in both rat model and human TBI patients, several biomarkers that were not significantly different between TBI and controls may also have significant correlation with white matter FA. As we suggested in our earlier publication (49), the variation in these biomarkers among TBI subjects may have simply reflected the inherent individual variation in the population and it was this individual variation that also contributed to the variation in TBI neurological outcomes as reflected by neuroimaging.

## 5 Conclusion

We found a general agreement in animal and human TBI findings that changes (usually increases) in plasma levels for the immunological biomarkers panel were associated with preservation of white matter integrity, as measured by diffusion MRI. These relationships were most apparent during the post-injury period where the animals or patients were clinically stable and at low risk of mortality and morbidity. We suppose that immunological reactions that were seemingly beneficial for white matter integrity in the present study might have been associated with negative outcomes in earlier studies in patients at risk for especially poor outcome.

## Data availability statement

The raw data supporting the conclusions of this article will be made available by the authors, without undue reservation.

## Ethics statement

The studies involving humans were approved by institutional Human Research Ethics Committee (approval number HREC/16/QRBW/604). The studies were conducted in accordance with the local legislation and institutional requirements. The participants provided their written informed consent to participate in this study. The animal study was approved by Animal Research Ethics Committee (AEC) of the University of Queensland (approval number: QBI/036/16/MAIC). The study was conducted in accordance with the local legislation and institutional requirements.

## Author contributions

XVT: Data curation, Formal Analysis, Methodology, Resources, Software, Visualization, Writing – original draft, Writing – review & editing. AM: Conceptualization, Investigation, Methodology, Resources, Writing – review & editing. PC: Writing – review & editing. FN: Conceptualization, Methodology, Project administration, Supervision, Writing – review & editing.
